# Editorial: Micronutrients and metabolic diseases - volume II

**DOI:** 10.3389/fnut.2025.1758663

**Published:** 2026-01-12

**Authors:** Jinhui Li, Aimin Yang, Yongting Luo, Peng An

**Affiliations:** 1 Department of Civil and Environmental Engineering, The Hong Kong Polytechnic University, Kowloon, Hong Kong SAR, China; 2 Department of Medicine and Therapeutics, The Chinese University of Hong Kong, Prince of Wales Hospital, Hong Kong, Hong Kong SAR, China; 3 Department of Nutrition and Health, China Agricultural University, Beijing, China

**Keywords:** metabolic disease, micronutrient, mineral, nutrition, vitamin

Healthy dietary patterns are recommended as preventive or therapeutic approaches for metabolic diseases (1). Healthy dietary patterns include abundant vegetables, fruits, and whole grains, and are rich in micronutrients such as antioxidant vitamins and minerals (2). Suboptimal intake of these micronutrients adversely affects glucose and lipid metabolism, increasing the risk of metabolic diseases, such as cardiovascular disease (CVD) and type 2 diabetes mellitus (3). Targeted dietary supplementation with certain micronutrients has been shown to improve cardiometabolic risk factors and health outcomes in at-risk populations (4).

The previous Research Topic, “*Micronutrients and metabolic diseases – Volume I*,” consisted of a series of articles focusing on the mechanisms of micronutrient metabolism and the relationship between micronutrients and human health (5). The current “*Micronutrients and metabolic diseases – Volume II*” extends this scope with of 14 new studies that investigate the influence of dietary patterns rich in micronutrients and the status of specific vitamins and minerals on metabolic diseases across diverse populations.

Adequate intake of B vitamins is critical for the maintenance of cardiometabolic health, especially the supplementation of B vitamins involved in homocysteine metabolism, which has been proposed as an effective strategy for the prevention of atherosclerotic cardiovascular diseases (6). In the current issue, three articles focus on the relationship between B vitamins and metabolic disease. Li H. et al. reported that dietary vitamin B1 intake was inversely associated with the risk of severe abdominal aortic calcification in a dose-response manner. Overton et al. reviewed the role of vitamin B_1_ in human health and the underlying mechanisms behind the therapeutic efficacy of thiamine in gastrointestinal diseases. Jiang et al. performed a Two-Sample Mendelian Randomization screening to reveal the causal relationship between micronutrients/serum metabolites and intervertebral disk degeneration. Among 15 micronutrients and 1,091 blood metabolites, they found that vitamin B_12_ displayed a negative correlation with the incidence of intervertebral disk degeneration, and 4-acetaminophen sulfate may act as a potential mediator of the protective effect of vitamin B_12_.

Metabolic dysfunction-associated fatty liver disease (MAFLD) affects approximately one-third of the global population. Individuals with MAFLD exhibit an elevated risk of other metabolic diseases (7). Three articles investigated the associations of diet patterns or a specific dietary micronutrient on the incidence of MAFLD (Yue et al., Lu et al., Tao et al.). Yue et al. observed that dietary antioxidant micronutrient vitamin E was negatively associated with the incidence of MAFLD, suggesting a contributing role of inflammation in the development of MAFLD. Tao et al. and Lu et al. additionally explored the influence of dietary or tissue inflammatory index on MAFLD.

Dysregulated glucose and lipid metabolism is the underlying pathological mechanism for most metabolic diseases. The triglyceride-glucose index has emerged as a novel tool in evaluating insulin resistance and metabolic diseases. Based on the United State National Health and Nutrition Examination Survey (NHANES) data, two studies, respectively, reported that vitamin D or zinc status correlated with the triglyceride-glucose index, indicating that nutrient status could aid in assessing insulin resistance (Lai et al.) or insulin resistance-related mortality risk (Zhang and Li). A systematic review by Laurindo et al. concluded that intake of polyunsaturated fatty acids-enriched seed oils had a positive impact on lipid profiles, blood glucose, and oxidative stress markers in patients with diabetes and dyslipidemia. These findings support the integrating of these nutrients into personalized nutrition strategies for the management of metabolic health.

In addition to studies focusing on specific nutrients, several articles in current issue examined the impact of dietary patterns on cardiometabolic health. Oxidative balance score (OBS) indicates the equilibrium between prooxidants and antioxidants in an individual's diet or lifestyle. Jin et al. reported that higher dietary or lifestyle OBS was associated with a reduced atherosclerotic cardiovascular disease risk, suggesting that a dietary pattern or lifestyle promoting more prooxidants may help mitigate the development of cardiovascular disease. Li S. et al. found that body mass index was involved in the preventative effect of the DASH diet on metabolic-related diseases. LiYa et al. reported that nutritional status affected gut microbiota, influencing disease progression and survival outcomes in esophageal cancer patients, and that low intake of carbohydrates and fiber was closely associated with malnutrition in these patients.

By spanning diverse populations and adopting rigorous methods, these articles advance our knowledge of the association between micronutrients and multiple domains of human health. As metabolic diseases continue to pose a worldwide health challenge, these findings provide practical insights to improve personalized nutrition strategies, ultimately aiding long-term efforts to lower the incidence of metabolic issues diseases.

## References

[1] Lichtenstein AH Appel LJ Vadiveloo M Hu FB Kris-Etherton PM Rebholz CM . 2021 Dietary Guidance to Improve Cardiovascular Health: A Scientific Statement From the American Heart Association. Circulation. (2021) 144:e472–87. doi: 10.1161/CIR.0000000000001031 34724806

[2] Belardo D Michos ED Blankstein R Blumenthal RS Ferdinand KC Hall K . Practical, Evidence-Based Approaches to Nutritional Modifications to Reduce Atherosclerotic Cardiovascular Disease: An American Society For Preventive Cardiology Clinical Practice Statement. Am J Prev Cardiol. (2022) 10:100323. doi: 10.1016/j.ajpc.2022.100323 35284849 PMC8914096

[3] Berger MM Shenkin A Schweinlin A Amrein K Augsburger M Biesalski H-K . ESPEN micronutrient guideline. Clin Nutr. (2022) 41:1357–424. doi: 10.1016/j.clnu.2022.02.015 35365361

[4] An P Wan S Luo Y Luo J Zhang X Zhou S . Micronutrient Supplementation to Reduce Cardiovascular Risk. J Am Coll Cardiol. (2022) 80:2269–85. doi: 10.1016/j.jacc.2022.09.048 36480969

[5] An P Yang A Li J Luo Y. Micronutrients and Metabolic Diseases. Front Res Top (2024) doi: 10.3389/978-2-8325-4671-0 PMC10967947 38544755

[6] Liu S An P. Untangling the uncertainty in B vitamins for stroke prevention: folic acid fortification, dosage, and their interaction? Am J Clin Nutr. (2024) 119:593–4. doi: 10.1016/j.ajcnut.2024.01.005 38432712

[7] Chan W-K Chuah K-H Rajaram RB Lim L-L Ratnasingam J Vethakkan SR. Metabolic Dysfunction-Associated Steatotic Liver Disease (MASLD): A State-of-the-Art Review. J Obes Metab Syndr. (2023) 32:197–213. doi: 10.7570/jomes23052 37700494 PMC10583766

